# Loss of Diacylglycerol Kinase α Enhances Macrophage Responsiveness

**DOI:** 10.3389/fimmu.2021.722469

**Published:** 2021-11-05

**Authors:** Laryssa C. Manigat, Mitchell E. Granade, Suchet Taori, Charlotte Anne Miller, Luke R. Vass, Xiao-Ping Zhong, Thurl E. Harris, Benjamin W. Purow

**Affiliations:** ^1^ Department of Pathology, School of Medicine, University of Virginia, Charlottesville, VA, United States; ^2^ Department of Pharmacology, School of Medicine, University of Virginia, Charlottesville, VA, United States; ^3^ Department of Neurology, Division of Neuro-Oncology, University of Virginia, Charlottesville, VA, United States; ^4^ Division of Allergy and Immunology, Department of Pediatrics, Duke University Medical Center, Durham, NC, United States

**Keywords:** DGKα, diacylglycerol kinase, macrophage, immune regulation, BMDM, wound healing

## Abstract

The diacylglycerol kinases (DGKs) are a family of enzymes responsible for the conversion of diacylglycerol (DAG) to phosphatidic acid (PA). In addition to their primary function in lipid metabolism, DGKs have recently been identified as potential therapeutic targets in multiple cancers, including glioblastoma (GBM) and melanoma. Aside from its tumorigenic properties, DGKα is also a known promoter of T-cell anergy, supporting a role as a recently-recognized T cell checkpoint. In fact, the only significant phenotype previously observed in *Dgka* knockout (KO) mice is the enhancement of T-cell activity. Herein we reveal a novel, macrophage-specific, immune-regulatory function of DGKα. In bone marrow-derived macrophages (BMDMs) cultured from wild-type (WT) and KO mice, we observed increased responsiveness of KO macrophages to diverse stimuli that yield different phenotypes, including LPS, IL-4, and the chemoattractant MCP-1. Knockdown (KD) of *Dgka* in a murine macrophage cell line resulted in similar increased responsiveness. Demonstrating *in vivo* relevance, we observed significantly smaller wounds in *Dgka^-/-^* mice with full-thickness cutaneous burns, a complex wound healing process in which macrophages play a key role. The burned area also demonstrated increased numbers of macrophages. In a cortical stab wound model, *Dgka^-/-^* brains show increased Iba1^+^ cell numbers at the needle track versus that in WT brains. Taken together, these findings identify a novel immune-regulatory checkpoint function of DGKα in macrophages with potential implications for wound healing, cancer therapy, and other settings.

## Introduction

The diacylglycerol kinases (DGKs) are the molecular switches regulating diacylglycerol (DAG) conversion to phosphatidic acid (PA). These enzymes have essential roles in lipid synthesis, cell proliferation, and survival, with DAG and PA playing key roles in regulating numerous critical enzymes and in recruitment of cytosolic proteins to the cell membrane. The protein kinase C (PKC) family of enzymes are key mediators of DAG effects, and are known to regulate cellular functions including proliferation, cell survival, and migration (1). In addition to being important regulators of a diverse set of cellular processes, PKCs have also been linked to tumorigenesis (2, 3). PA is also able to influence processes such as cell proliferation through its binding of the FKBP12-rapamycin binding region of mTOR (4, 5). Therefore, proper regulation of DGK enzymes is crucial for maintaining homeostasis of DAG, PA, and their downstream effectors.

The ten mammalian DGKs are divided into five subtypes based on shared functional domains. All ten DGKs possess both a C1 and a catalytic domain, with variations existing in the N- and C-terminal regions. Even within subtypes, the DGKs may differ in abundance and in organ/subcellular distribution. For instance, Type I DGKs α and β differ markedly in tissue distribution; DGKα is one of the most commonly expressed DGKs and particularly enriched in the brain, lungs, and white blood cells, while DGKβ is found at lower levels and predominantly in nerves and the brain (5, 6). This suggests potential specificity of certain DGKs for different cell types, and the occurrence of individual DGKs from different subtypes within a single tissue suggests their having specific functions. One known and recently identified function of DGKα – along with its other family member DGKζ – is its promotion of T-cell anergy (7–9). The ability to limit T-cell activation in the tumor microenvironment, along with the promotion of tumor survival resulting from PA generation (10), are a tandem that underlie the challenges of therapeutic development in cancer, and present an opportunity for targeting DGKα in this context. The notion of DGKα as an immune regulator has become firmly established, with it now being described as an immune checkpoint (11), and its inhibition has been proposed as an approach for improving CAR-T cell performance (12). A role in regulating NK cell function has also been shown, with restoration of disabled MAPK pathway function in NK cells from human renal cell carcinoma following DGKα inhibition (13). However, a role for DGKα in macrophage function and the innate arm of the immune system has never been demonstrated.

Macrophages are important effector cells of the innate immune system and perform many varied and context-dependent functions. They are crucial to the maintenance of tissue homeostasis, and are among the first cells to respond in the event of injury (14). In addition to their important role in host defense, they also are necessary for tissue remodeling and repair. Pro-inflammatory macrophages are known to exhibit increased secretion of pro-inflammatory cytokines, and sometimes increased phagocytosis, aiding in the removal of pathogens and damaged tissue. Anti-inflammatory macrophages secrete high levels of anti-inflammatory cytokines and fibrogenic and angiogenic factors that promote tissue remodeling and repair (15). These polarization states have relatively well-defined stimuli and markers, which promote the state of activation best suited for the current environmental condition. Lipopolysaccharide (LPS) typically induces a pro-inflammatory phenotype, promoting upregulation of genes associated with inflammation, tumor resistance, and graft rejection, while IL-4 promotes matrix deposition necessary for tissue remodeling and repair, characteristic of an anti-inflammatory phenotype (16). iNOS and Arginase-1 are two commonly referenced markers of pro- and anti-inflammatory activation, respectively. They play important roles in promoting the conditions which give rise to their respective polarization states (17). In the tightly regulated process of wound healing, pro-inflammatory macrophages are particularly important during the first inflammatory phase, while anti-inflammatory macrophages can be found during the proliferative and remodeling phases.

While DGKα has not been firmly linked to the innate arm of the immune system, we came to suspect such a connection following the observation that there were dramatically fewer skin ulcers over subcutaneous melanoma tumors in *Dgka^-/-^* versus wild-type (WT) mice. Given that DGKα had been shown to regulate other immune cells and that macrophages are known to play key roles in wound healing (18), including in the context of chronic wounds such as skin ulcers (14), we hypothesized that DGKα regulates macrophage activity as well. This prompted us to specifically investigate macrophage function in the setting of *Dgka* knockout and knockdown. Through these experiments, we have identified a novel role for DGKα in macrophage responsiveness and activation, using bone marrow-derived macrophages (BMDMs), a murine macrophage line, and two *in vivo* injury models.

## Materials & Methods

### BMDM Harvest

BMDMs were derived from bone marrow harvested from 12-week old WT and *Dgka^-/-^* C57BL/6 mice. Mice were euthanized, hind limbs excised, and then skin, muscle, and connective tissue removed. Bare femur was carefully separated from tibia/fibula, then both ends of each bone clipped to expose bone marrow. Using a 19G needle, a small hole was poked through the bottom of a 0.5mL Eppendorf tube which was then placed inside a 1.5mL Eppendorf tube. Clipped bones were inserted into the 0.5mL tube and the nested tubes spun for 10-20 seconds in a mini centrifuge, drawing marrow through the needle hole into the 1.5mL tube. Red blood cell progenitor lysis was accomplished with 0.83% (w/v) NH_4_Cl for 5 minutes at room temperature. Pelleted marrow cells were resuspended in BMDM culture medium.

### Macrophage Differentiation and Activation

BMDM culture medium was prepared with RPMI 1640 with 2mM L-glutamine, 10% FBS, 2% HEPES, and 2% antibiotic-antimycotic. Differentiation medium was prepared as 90% BMDM culture medium and 10% L929 conditioned medium (LCCM) containing macrophage colony stimulating factor (M-CSF). BMDMs were cultured for four days in differentiation medium, which was replaced on days four and six. Differentiated macrophages were plated on day seven in BMDM culture medium without LCCM and incubated overnight followed by treatment with BMDM medium supplemented with 500ng/mL LPS (Invitrogen, #00-4976-93) or 2ng/mL IL4 (BioLegend, #574302). Cells were lysed for western blot and qPCR analysis.

### Migration Assay

Following differentiation of BMDMs, cells were starved in FBS-free media for 24h. After trypsinization and counting, 3x10^5^ cells were plated into 8μm transwell inserts purchased from Abcam (#235694). The wells below transwells contained 600μL of culture media with 50ng/mL of monocyte chemoattractant protein-1 (MCP-1). Eighteen hours after the start of the assay, migrated cells were dissociated from the underside of the transwell and quantified per the manufacturer’s instructions.

### Immunoblot

Western blots were done on BMDM lysates using Cell Signaling Technology Cell Lysis Buffer 10x (#9803) supplemented with 0.5% SDS and protease inhibitor tablet (Roche, #04693124001). ~5μg protein was loaded and PVDF membranes were probed with antibodies specific to iNOS (Novus Biologicals, #NB300-605), Arginase-1 (Proteintech, #16001-1-AP), β-tubulin (Proteintech, #10094-1-AP), β-actin (Thermo Fisher Scientific, # PA5-85291), DGKα (Proteintech, #11547-1-AP), and Phospho-(Ser) PKC Substrate (Cell Signaling, #2261). Quantification of immunoblots was performed by first normalizing band intensities to β-tubulin or β-actin to control for loading variability. Relative intensities were then calculated by normalizing to PBS control conditions.

### Real-Time qPCR

RNA isolation was performed using Qiagen RNeasy Mini Kit (#74104), and cDNA synthesis done with Qiagen QuantiTect Reverse Transcription kit (#205311) according to manufacturer’s instructions. Real-time qPCR was performed using Applied Biosystems PowerSYBR Green PCR Master Mix (#4367659). Expression of target mRNA was normalized to ribosomal 18s RNA and quantified using the 2^-ΔΔCT^ method. Primer pairs were sourced from the PrimerBank of the Center for Computational and Integrative Biology of Massachusetts General Hospital and Harvard University (19–21). Sequences used were Mouse 18s Forward: GTAACCCGTTGAACCCCATT, Reverse: CCATCCAATCGGTAGTAGCG; iNOS (PrimerBank ID 146134510c1) Forward: GTTCTCAGCCCAACAATACAAGA, Reverse: GTGGACGGGTCGATGTCAC; Arginase-1 (PrimerBank ID 158966684c1) Forward: CTCCAAGCCAAAGTCCTTAGAG, Reverse: GGAGCTGTCATTAGGGACATCA; SOCS1 (PrimerBank ID 6753424a1) Forward: CTGCGGCTTCTATTGGGGAC, Reverse: AAAAGGCAGTCGAAGGTCTCG; SOCS3 (PrimerBank ID 6671758a1) Forward: ATGGTCACCCACAGCAAGTTT, Reverse: TCCAGTAGAATCCGCTCTCCT. Ki67 (PrimerBank ID 1177528a1) Forward: ATCATTGACCGCTCCTTTAGGT, Reverse: GCTCGCCTTGATGGTTCCT. DGKα (PrimerBank ID 31560473c1) Forward: GTGATGTGTACTGCTACTTCACC, Reverse: CACTTCCGTGCTATCCAGGA. DGKβ (PrimerBank ID 26336555a1) Forward: ATGAAGACCTTTCTGGAAGCTG, Reverse: TTTACATTTGGGCTAGAATGGGG. DGKγ (PrimerBank ID 26354382a1) Forward: ATGAGCGAAGAACAATGGGTC, Reverse: GGGCTTGTGTGGGTCATACTG. DGKζ (PrimerBank ID 30794244a1) Forward: CTCTTTGGGCACAGGAAAGC, Reverse: TGCTGACTCACTCCAGTCCA.

### Cell Culture and Transfection

J774 macrophages were cultured in DMEM supplemented with 10% FBS and 1% penicillin-streptomycin. Cells were transfected using Lipofectamine RNAiMAX (Invitrogen, #13778030) with control (Ambion #AM4611) and DGKα siRNA (Ambion, #AM16708 – 161793, 161794).

### Mouse and Wound Models

The *Dgka^-/-^* mouse colonies used in these experiments were established in the lab of Dr. Xiao-Ping Zhong at Duke University, and were generated as previously described (9). Cutaneous burn model mice were anesthetized using ketamine/xylazine. Hair was removed by shaving, followed by depilatory cream applied for 3 minutes. An 8mm-diameter metal rod set in boiling water was then applied for 10 seconds to bare skin raised from both flanks. Mice were then given buprenorphine slow-release analgesic, rehydrated, and returned to single housed cages. Burns were photographed daily using isoflurane anesthesia and imaged by microscope equipped with an Amscope MD200 camera. Measurements were taken using ImageJ image processing software.

Cortical stab wounds were performed by stereotactic alignment and immersion of a 30G needle 2mm lateral and 1mm anterior to the bregma, advancing 3mm into the brain. Brains were saline-perfused and harvested four days post-injury, then formalin-fixed in 10% formalin for 24h at room temperature, then transferred to 70% ethanol prior to paraffin-embedding. All *in vivo* experiments were performed under IACUC-approved protocol 3833.

### Immunohistochemistry

Mouse skin and brains were fixed in 10% formalin for 24h at room temperature, then transferred to 70% ethanol prior to paraffin embedding. Immunohistochemistry was performed on a robotic platform (Ventana discover Ultra Staining Module, Ventana Co., Tucson, AZ, USA). Tissue sections (4 µm) were deparaffinized using EZ Prep solution (Ventana). A heat-induced antigen retrieval protocol set for 64 min was carried out using Cell Conditioner 1 (Ventana). Endogenous peroxidases were blocked with peroxidase inhibitor (CM1) for 8 min before incubating the section with Iba1 antibody (Invitrogen, Cat#PA5-21274) at 1:1,250 dilution for 60 min at room temperature. Antigen-antibody complex was then detected using DISCOVERY OmniMap anti-rabbit multimer RUO detection system and DISCOVERY ChromoMap DAB Kit (Ventana Co.). All slides were counterstained with hematoxylin subsequently; they were then dehydrated, cleared and mounted for assessment.

### Image Analysis

Whole slide images of immunohistochemical slides were obtained on the Aperio ScanScope slide scanner at 20x magnification. All images were analyzed using the open source digital pathology software, QuPath (v0.2.3) (22). In the software, stained cells were quantified as number positive/mm^2^ and segmented by size and shape, with a range of 50-500μm^2^ to capture cells of varying depths within the plane.

### Immunofluorescence

Manual immunofluorescent (IF) staining was done on mouse brain and skin slides blocked for one hour at room temperature (RT) in a humidity chamber with 5% mouse serum, 5% donkey serum, and 0.3% Triton X-100. Primary antibody solutions were prepared in PBS with 0.3% Triton X-100. Primary antibodies used were specific to F4/80 (Novus Biologicals, #NB800-404), Iba1 (Novus Biologicals, #NB100-1028), and DGKα (Bioss Antibodies, #BS-14294R). Tissue was incubated overnight at 4°C in humidity chamber. Fluorescent antibodies (Abcam) used were donkey anti-rat AF647 (#ab150155), donkey anti-rat AF488 (#ab150153), donkey anti-rabbit AF647 (#ab150075), and donkey anti-goat AF488 (#ab150133). Secondary solutions were prepared in PBS with 0.3% Triton X-100 and incubated on slides for three hours at RT in humidity chamber protected from light. Stained slides were mounted using mounting medium with DAPI (Abcam, #ab104139). Slides were imaged using the Leica Thunder Imager at 10x magnification, and analyzed on the Leica Application Suite X (LAS X) software platform.

### Statistics

Statistical analysis was performed using GraphPad Prism 8 software. Student’s t test (two-tailed) was used for analysis of differences between two groups. One or two-way ANOVA with Tukey or Sidak post-hoc test was used for multiple comparisons. Data are presented as mean ± SD. P < 0.05 was considered statistically significant.

## Results

### Dgka^-/-^ BMDMs Are More Responsive to LPS and IL-4 Stimulation

**Figure 1:**
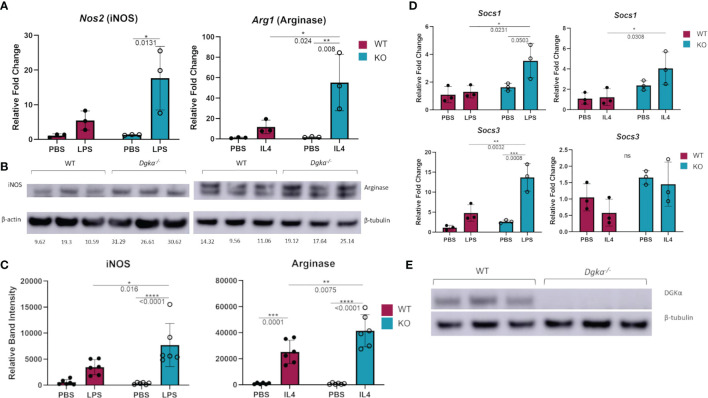
Increased expression of macrophage activation markers in *Dgka* ^-/-^ BMDM following LPS or IL4 stimulation. **(A)** WT and *Dgka^-/-^* BMDMs were treated with 500ng/mL LPS or 2ng/mL IL-4 for 24h and assessed by qPCR for changes in *Nos2* and *Arg1* expression, respectively. **(B)** Immunoblot depicting protein expression of iNOS in lysates of LPS treated WT and *Dgka^-/-^* BMDMs (left) and arginase expression of IL-4 treated BMDMs. Quantification of band intensity normalized to PBS treated WT mice (not shown) are indicated in text below bands. **(C)** Summary plot depicting quantification of immunoblot band intensity of two independent experiments as in **(B)** for n = 6 per group. **(D)** WT and *Dgka^-/-^* BMDMs treated with LPS and IL-4 were assessed by qPCR for expression of SOCS1 and SOCS3. In **(A–D)** samples were normalized to the PBS treated WT control. **(E)** immunoblot of WT and *Dgka^-/-^* BMDM whole cell lysates with anti-DGKα antibody. Each data point represents an individual mouse for n = 3 per group. Error bars: SD. *p < 0.05, **p < 0.01, ***p < 0.001, ****p < 0.0001; ns, not significant; Two-way ANOVA with Tukey post-hoc test.

To test whether DGKα regulates macrophage activity and responsiveness, we isolated and cultured BMDMs from *Dgka^-/-^* and WT mice and tested the expression of activation markers upon stimulation with LPS or IL-4. qPCR analysis demonstrated that *Nos2* (gene encoding iNOS) expression was significantly increased 24h after LPS stimulation, as was *Arg1* (gene encoding arginase) expression following IL-4 stimulation (**Figure 1A**). Differences are evident both as a direct contrast between *Dgka^-/-^* and WT, and also in the degree of upregulation from baseline with PBS control-treated cells. These differences were also evident in the immunoblot analysis of WT and *Dgka^-/-^* BMDM lysates at 24h post stimulation (**Figures 1B, C**). We additionally tested the expression of two members of the SOCS family, which are intracellular cytokine-inducible proteins known to regulate macrophage activation and polarization (23). While SOCS1 is reported to promote both pro- and anti-inflammatory activation, SOCS3 has been found to have a key role in pro-inflammatory polarization alone (24, 25). Consistent with these roles, we have found in BMDMs that *Socs1* expression responds strongly to LPS and IL-4 stimulation, and that *Socs3*, expression is enhanced only by LPS stimulation – with both showing greater increases in *Dgka^-/-^* BMDMs than in WT BMDMs (**Figure 1D**). To confirm the genotype of study mice use, BMDM lysates were also probed for DGKα protein expression (**Figure 1E**). Interestingly, we observed that DGKα expression is affected by both LPS and IL-4 in WT BMDMs (**Supplementary Figures 1A, B**). An increase in DGKα protein expression was evident in LPS-treated WT BMDMs, while IL-4 treatment resulted in reduced protein levels (**Supplementary Figure 1C**). Given that there are ten DGK family members including DGKα, we considered whether other DGKs compensate for its absence in treated and untreated conditions (**Supplementary Figure 1D**); we found that loss of DGKα caused no change in mRNA expression levels of the remaining type I DGKs, nor type IV DGKζ. It was not surprising to observe the presence of DGKα transcript within the *Dgka^-/-^* samples given the KO strategy, as this KO does still yield a modified *Dgka* transcript, but not the protein.

### Increased Responsiveness of J774 Macrophages to LPS and IL-4 Stimulation and Increased PKC Activity Following Dgka Knockdown

**Figure 2:**
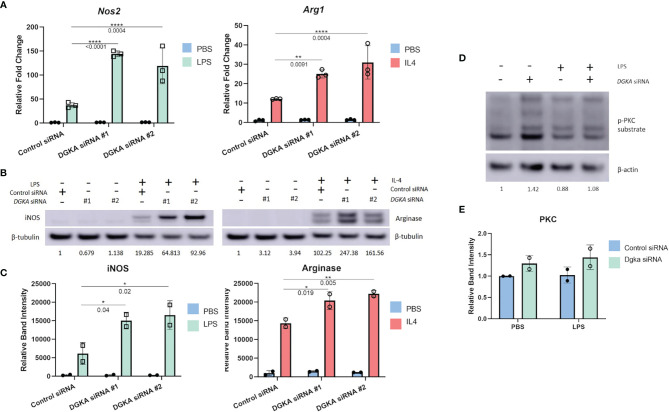
Knockdown of *Dgka* from J774 murine macrophage cell line shows increased amplification of macrophage activation markers following LPS or IL4 stimulation. **(A)** J774 murine macrophages were stimulated with 500ng/mL LPS or 2ng/mL IL-4 following control or *Dgka* siRNA transfection and assessed by qPCR for *Nos2* and *Arg1*, respectively. Samples were normalized to the PBS treated control siRNA transfected cells. **(B)** Immunoblot analysis of whole cell J774 lysates probing for iNOS in LPS-treated cells and Arginase in IL4-treated cells. Relative band intensities normalized to PBS-treated control are indicated as text below the immunoblots. Representative data shown. **(C)** Summary plot depicting quantification of immunoblot band intensity from two independent experiments as in **(B)**. **(D)** J774 macrophages transfected with either control siRNA or *Dgka* siRNA were treated with LPS for 24h, and the protein expression of phospho-PKC substrates is shown by immunoblot. Relative band intensities normalized to PBS treated control are indicated below the immunoblots. **(E)** Summary plot depicting quantification of immunoblot band intensity of two independent experiments as in **(D)**. Error bars: SD. *p < 0.05, **p < 0.01, ****p < 0.0001; Two-way ANOVA with Tukey post-hoc test.

We tested whether *Dgka* knockdown would similarly increase responsiveness to cytokine stimulation in a widely-used murine macrophage line. In the J774 line, transient transfection with two different *Dgka* specific siRNAs indeed resulted in similar patterns of macrophage marker upregulation by qPCR. Following LPS and IL-4 stimulation, J774 cell expression of *Nos2* and *Arg1*, respectively, was increased in cells that had been transfected with *Dgka* siRNA significantly more than in cells transfected with negative control siRNA (**Figure 2A**). In the absence of LPS or IL-4 stimulation, mRNA expression levels are the same across groups. Western blot analysis of whole cell lysates further supported this increased stimulation (**Figure 2B**). Quantification of increases in iNOS and Arginase protein expression are also shown (**Figure 2C**). We also noted more prolonged iNOS protein expression in LPS-treated *Dgka* KD cells over a time course of 24h compared to control siRNA-transfected cells (**Supplementary Figures 2A, B**).

As previously noted, PKC is a key effector of the DGKα substrate DAG. To determine if downstream DAG signaling in the absence of DGKα influences PKC, and therefore implicate PKC as potential mediator of increased *Dgka* KD macrophage responsiveness, an immunoblot of the *Dgka* siRNA transfected J774 lysates was probed for phosphorylated PKC substrates (**Figures 2D, E**). 48h after siRNA transfection, and 24h after PBS or LPS treatment, PBS-treated J774 cells with *Dgka* KD exhibit increased levels of phospho-PKC substrates versus control siRNA-transfected J774. While LPS treatment did not demonstrate an increase in PKC activation, LPS-treated cells that were transfected with *Dgka* siRNA exhibited modestly higher levels of phospho-PKC substrates than control siRNA-transfected cells. Successful *Dgka* knockdown was confirmed by western blot (**Supplementary Figures 3A, B**).

### Increased Sensitivity to Regulation of Cell Cycling With Dgka Knockout/Knockdown

**Figure 3:**
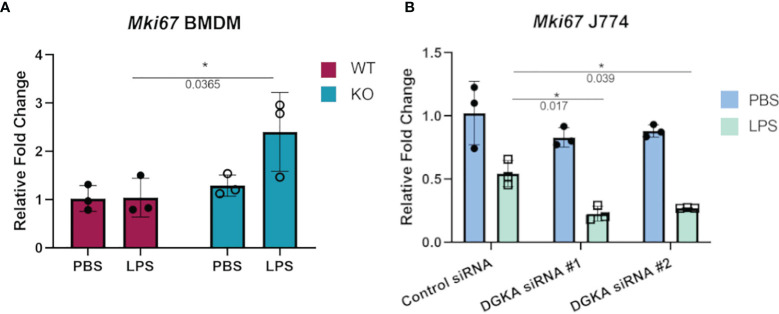
Increased sensitivity to regulation of cell cycling with *Dgka* knockout/knockdown. **(A)** qPCR analysis of *Mki67* expression in WT and *Dgkα^-/-^* BMDMs treated for 24h with 500ng/mL LPS. **(B)** J774 macrophages transfected with two different *Dgka* siRNAs were similarly assessed by qPCR for *Mki67* following LPS stimulation. Each data point in **(A)** represents an individual mouse for n = 3 per group. Representative data are shown in **(B)**. Samples were normalized to PBS-treated WT or control siRNA cells. Error bars: SD. *p < 0.05; Two-way ANOVA with Tukey post-hoc test.

Macrophages also respond to some stimuli with increased cell cycling, and proliferation of tissue-resident macrophages has been identified as a response to local inflammation and injury (26, 27). We therefore tested whether BMDMs and J774 would have increased expression of a widely-used marker of cell cycling in response to LPS stimulation with *Dgka* knockout and knockdown, respectively. Ki67 expression, which has long been used in immunohistochemical staining as a marker of cell proliferation, has also been measured by qPCR to measure cell cycling (28–30). In differentiated and minimally proliferative BMDMs, only *Dgka^-/-^* cells had an increase in *Mki67* expression by qPCR in response to LPS stimulation (**Figure 3A**). In contrast, it has been reported that LPS treatment of J774 cells inhibits their proliferation (31, 32). We indeed observed this in J774 cells, as indicated by a decrease in *Mki67* expression by qPCR, and found that *Dgka* knockdown increased sensitivity to this inhibition – supporting our hypothesis of increased macrophage responsiveness, albeit in this case with a negative response (**Figure 3B**). In the interest of assessing the proliferative potential of LPS-treated BMDMs, WT macrophages were treated for 48h with PBS or 500ng/mL of LPS and counted (**Supplementary Figure 4**). We found that there was indeed a significant increase in cell number in the LPS-treated condition. BMDMs have a tendency to be more proliferative than macrophages derived from other sources (33), and may thus be at the proliferative end of the spectrum of variable macrophage responses to LPS.

### Increased Migration of Dgka^-/-^BMDMs *In Vitro*

**Figure 4:**
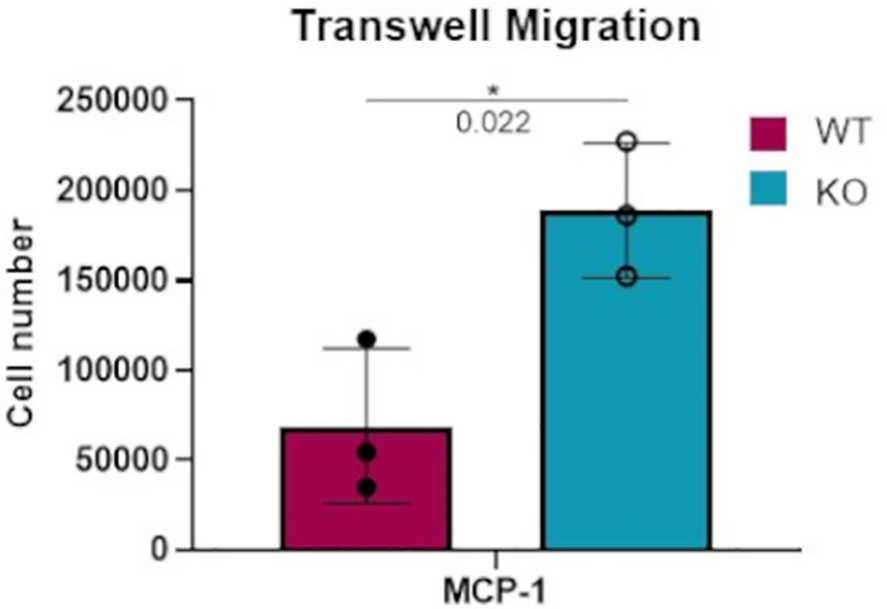
Increased transwell migration toward MCP-1 in *Dgka^-/-^* BMDMs. 3x10^5^ WT and *Dgka^-/-^* BMDMs were FBS-starved for 24h, then plated into 8μm pore transwells over media containing 50ng/mL MCP-1. After 18 hours, transwells were removed and cells were counted per the manufacturer’s instructions. Error bars: SD. *p < 0.05; Student’s t-test.

In considering the potential physiological effects of increased macrophage responsiveness resulting from DGKα loss, a transwell migration assay was performed to compare the responsiveness of WT versus *Dgka^-/-^* BMDMs to a chemoattractant. When FBS-starved cells were plated on an 8μm pore membrane transwell across from 50ng/mL MCP-1, a significant increase in cell migration was observed after 18h in *Dgka^-/-^* BMDMs compared to WT (**Figure 4**).

### Smaller Wounds From Full-Thickness Cutaneous Burns in Dgka^-/-^ Skin

**Figure 5:**
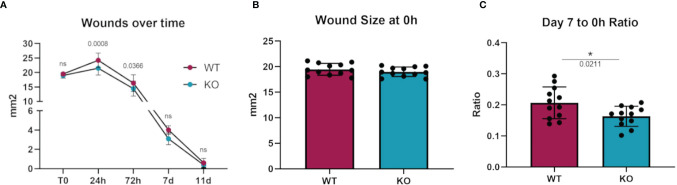
Increased wound closure rate in *Dgka* ^-/-^ mice compared to WT. **(A)** Area measurement of burn wounds in KO and WT mice compared over time. **(B)** Sizes of wounds from both groups are comparable at time zero. **(C)** One week post injury, wound sizes in KO mice are smaller relative to those at T0. Each data point represents a single burn, with two burns on the each of n = 6 mice per group. Error bars: SD. *p < 0.05; ns, not significant. Two-way ANOVA with Sidak post-hoc test **(A)**, Student’s t-test **(B, C)**.

To investigate whether an *in vivo* pathologic process dependent on macrophages would show differences in *Dgka^-/-^* mice, we assessed differences in wound healing with a full-thickness cutaneous burn model. When cutaneous burns were induced in the flanks of study mice, *Dgka^-/-^* mouse wounds were found to be smaller than those from WT mice at 24 and 72 hours (**Figure 5A**). Blinded wound area measurements were taken at specified time-points, and initial wound sizes were comparable across all mice (**Figure 5B**). The ratio of wound size at day seven to initial size was significantly smaller in the *Dgka^-/-^* mice, but was no longer different by day eleven when wounds were largely closed (**Figure 5C**). Wound area at 24 hours post injury increased from the initial area measurements due to expected and rapid tissue inflammation, but interestingly this increase in size was less pronounced in *Dgka^-/-^* mouse wounds.

### Increased Macrophage Numbers at Site of Skin Burn Injury in Dgka^-/-^ Mice

**Figure 6:**
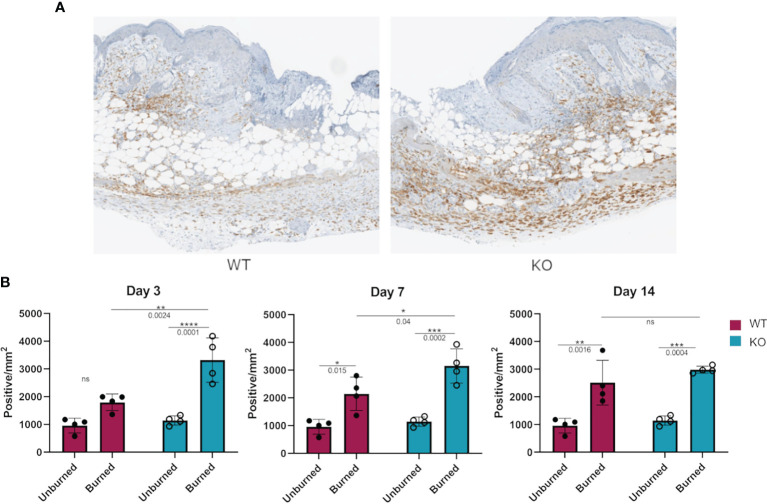
Increased numbers of Iba1^+^ macrophages in burned skin of *Dgka^-/-^* mice. **(A)** IHC staining of Iba1^+^ macrophages from excised and bisected day 3 wounds of WT and *Dgka^-/-^* mice show increased number of cells at the site of the burn. **(B)** Quantification of Iba1^+^ stained cells in burned skin at three time-points in comparison to unburned skin. Each data point represents a single burn, with two burns per mouse per time-point for n = 6 mice per group. Error bars: SD. *p < 0.05, **p < 0.01, ***p < 0.001, ****p < 0.0001; ns, not significant; Two-way ANOVA.

We hypothesized that the more responsive macrophages in *Dgka^-/-^* mice might be present in greater numbers at a site of tissue injury and inflammation. Flank skin was harvested from study mice from the experiments in **Figure 4** at days three, seven, and fourteen and processed for immunological staining for the macrophage marker Iba1 (**Figure 6A**). Additionally, skin from the flanks of unburned mice was also collected and similarly processed for comparison. Tissue images were annotated to analyze regions of the burn, defined as the full thickness of skin below eschar or open wound, epi/dermis (consisting of both the epidermis and dermis above the panniculus carnosus), and the hypodermis (the region below the panniculus carnosus). The number of Iba1^+^ stained cells in each region was compared between groups (**Supplementary Figure 5**). We found a significant increase in macrophage numbers in *Dgka^-/-^* burns at day three (**Figure 6B**). This included increased Iba1^+^ cells compared to both unburned *Dgka^-/-^* skin and burned WT skin. There was no significant difference in the number of Iba1^+^ cells between unburned and burned WT mice at day three. This changes by days seven and fourteen, when we observed the number of Iba1^+^ cells increase in WT skin over time and catch up to numbers in the *Dgka^-/-^* skin burn area (which remained stable). This may correlate with the prominent role of macrophages in early wound healing (18). Supporting the validity of Iba1 as a macrophage marker for skin, as well as confirming expression of DGKα in macrophages, we performed immunofluorescence showing colocalization of Iba1 plus the mouse macrophage marker F4/80 or DGKα plus F4/80 in mouse skin (**Supplementary Figures 6A, B**).

### Increased Iba1^+^ Cell Infiltration at the Site of Cortical Stab Injury in Dgka^-/-^ Brains

**Figure 7:**
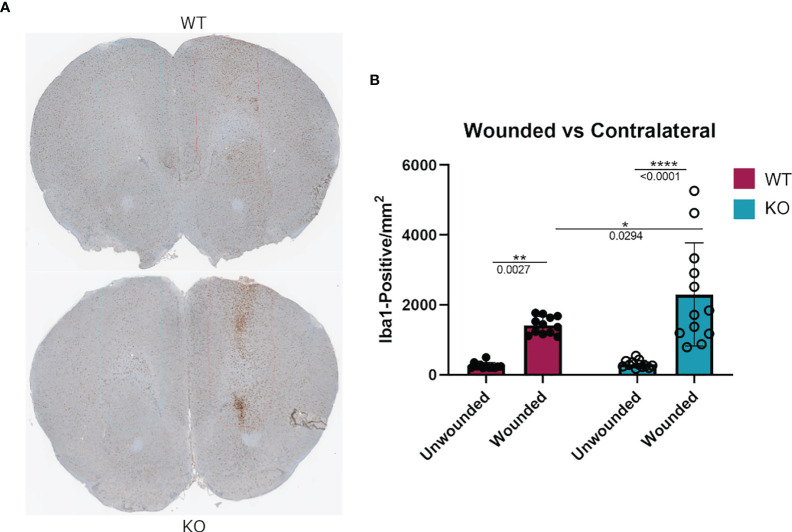
Increased Iba1^+^ cell infiltration at needle track of surgically wounded *Dgka^-/-^* and WT mice in acute brain injury model. **(A)** Representative cross sections highlighting needle track and unwounded contralateral cortex. **(B)** Quantification of Iba1^+^ cells in the needle track demonstrates an increase in cell infiltration in wounded *Dgka^-/-^* brains versus that in the contralateral, unwounded side. Each data point represents one of four brain sections cut from the site of needle injection, from each of n = 3 mice per group. Error bars: SD. *p < 0.05, **p < 0.01, ****p < 0.0001; Two-way ANOVA with Tukey post-hoc test.

To assess if the phenomenon of DGKα loss resulting in increased macrophage responsiveness extended to other *in vivo* injuries as well, we tested a cortical stab wound model in *Dgka^-/-^* versus WT mice. Microglia are resident macrophage analogs in the brain, and are the predominant Iba1^+^ cell population at this site other than in rare pathologies such as brain tumors. Activated microglia, which are induced in situations such as brain injury and inflammation, have higher expression of Iba1 than do resting microglia. Four days after a surgical brain injury with a needle, mouse brains were collected and subsequently sectioned and stained for Iba1^+^ cells. When each was compared to the uninjured contralateral brain, *Dgka^-/-^* wound areas showed significantly more Iba1^+^ cells in the region than in equal wound areas from WT mouse brains, as evident in representative brain slices (**Figure 7A**) and with quantification (**Figure 7B**). Immunofluorescence confirmed colocalization of DGKα and Iba1 in brain microglia (**Supplementary Figure 6C**).

## Discussion

With these studies, we have identified a novel mechanism by which DGKα regulates the immune system. Stimulation of BMDMs derived from WT and *Dgka^-/-^* mice with either LPS or IL-4 upregulated expression of macrophage activation markers in both groups, but consistently did so to a greater extent in the *Dgka^-/-^* mice. Baseline expression of activation markers was not enhanced by DGKα loss, but the macrophage response to stimulation was enhanced. Notably, this was not simply a shift in polarization, as the responses to both pro- and anti-inflammatory promoting stimuli were enhanced and expression of classic pro- and anti-inflammatory markers were each increased. Sensitivity of macrophages to diverse stimuli including LPS, IL-4, and the chemoattractant MCP-1 was increased in each case. Elevated expression of iNOS and arginase in response to stimuli could itself have significant implications, as each plays an important role in macrophage function. iNOS is implicated in pathogen scavenging through its production of NO and citrulline from arginine. By converting arginine to polyamine and collagen precursor ornithine, the enzyme arginase contributes to extracellular matrix production. Increasing expression of both in macrophages with the use of a DGKα inhibitor might promote host defense and tissue repair, in keeping with our results in the wound models. Confirming our findings in *Dgka* knockout mouse macrophages, transient knockdown of *Dgka* in a murine macrophage line yielded similar results. We were also able to observe an increase in PKC activation with *Dgka* knockdown, providing a potential mechanism for elevated macrophage responsiveness. We hypothesize that the increase in some DAG species with *Dgka* knockout/knockdown and the resulting increase in PKC activation boosts macrophage responsiveness to numerous stimuli, with PKC activity acting as a rheostat controlling macrophage stimulation. In support of this, PKC has been reported as a key element in macrophage cell signaling in response to diverse inputs (34–36).

The promotion of macrophage cell cycling in these same stimulatory conditions, as evidenced by increased Ki67 mRNA expression in *Dgka^-/–^* derived BMDMs, presents further support for enhanced cell responsiveness; this was evident even in terminally differentiated and minimally proliferative cells. In cells with suppressed proliferation, as is the case with LPS-treated J774 macrophages, *Dgka* knockdown led to further downregulation of *Mki67* than that observed in controls. These results are the first demonstration of increased responsiveness of macrophages with *Dgka* knockout and inhibition, as previous interactions between DGKα and immune activation have been limited to T-cells and NK cells (13). Importantly, increased responsiveness was demonstrated with pure macrophage cultures such as a cell line, indicating it was not a secondary effect due to increased activity of neighboring immune cells such as T or NK cells.

The brain injury model also indicated higher numbers of Iba1^+^ cells in the wound area of *Dgka^-/-^* mice. While not confirmed as activated microglia, this is likely given their predominance in the region of brain injury; the result therefore suggests that the effects of *Dgka* knockout may extend to tissue-resident macrophage analogs. Our burn model sought to highlight any differences this knockout might have in the wound healing process, and to identify the cells being regulated. Our initial observation of decreased skin ulceration over subcutaneous tumors in *Dgka^-/-^* mice not only prompted us to investigate macrophage involvement, but also suggested that the response to injury might be altered in *Dgka^-/-^* mice. Our findings in the burn model supported this, with smaller burn wounds and locally increased macrophage numbers in *Dgka^-/-^* mice. Our *in vitro* findings of increased cell cycling and increased migration of *Dgka* knockout/knockdown macrophages suggest that either increased proliferation or influx of these macrophages could explain their higher numbers at wound sites. Notably, our results relied on the use of Iba1 as a well-validated specific marker for macrophages and local macrophage analogs (37, 38). The specificity of Iba1 to the microglia in the brain has also been reported (39), and evidence that phenotypic Iba1 expression is comparable to that of CD68 and F4/80 was included in establishing this macrophage/microglia marker (40). The process of healing in mouse skin is extremely efficient, making it difficult to identify anything that improves wound healing or wound size. Macrophages are at the forefront of this rapid wound closure and healing in mouse skin, and increasing their numbers and responsiveness at wound sites could have substantial implications for healing. Both our wound models support the *in vivo* relevance of this work and its potential implications for tissue injury and healing.

The potential of DGKα as a therapeutic target in several cancers has been demonstrated by us and others, and more potent and specific DGKα inhibitors are in development at large pharmaceutical companies. While they are being developed as immunotherapy adjuncts to promote T and NK cell activity, our results suggest it will be critical to also determine the effects of these inhibitors on macrophages. Given that tumor-associated macrophages (TAMs) have powerful tumor-promoting effects (41, 42), it must be assessed whether DGKα inhibition promotes or suppresses this pro-tumor macrophage activity; these studies are ongoing. DGKα inhibition would be especially appealing if it were able to not only directly attack cancer cells and boost T and NK cell activity, but also improve macrophage activity against cancer cells.

While these findings extend the role of DGKα in the immune system, with potential clinical implications, we acknowledge significant limitations in this study. We have not definitively identified the molecular mechanism by which *Dgka* knockout/knockdown increases responsiveness of macrophages, though this is a subject of active investigation, and our current study has suggested elevated PKC activity as a potential mediator. The relevant signaling appears to differ from the primary reported mechanism for the T cell regulation by DGKα, which has been reported to include increased Ras activity (8). This study is also limited to murine macrophages and mice, and it is important to extend these findings to human macrophages.

Taken together, these data present a novel immunologic function for DGKα as a regulator of macrophage activation. This is the first demonstration of a DGK family member suppressing macrophage function; one prior report linked DGKζ to macrophage activity in juvenile arthritis and cytokine storm mouse models (43), but it showed a stimulatory role for DGKζ opposite that we observed for DGKα. If DGKα plays a similar role in human macrophages and related cells, this extends its recently-identified role as an immune checkpoint to the innate immune system and may also have therapeutic implications. As noted above, more potent and specific DGKα inhibitors are in development as adjuncts for cancer immunotherapy, and these findings could extend their utility beyond the cancer setting. Given the role of macrophages in healing injury and combating infectious disease, DGKα inhibitors might find clinical applications in treating these pathologies as well.

## Data Availability Statement

The original contributions presented in the study are included in the article/**Supplementary Material**. Further inquiries can be directed to the corresponding author.

## Ethics Statement

The animal study was reviewed and approved by the University of Virginia Institutional Animal Care and Use Committee (IACUC).

## Author Contributions

LM conceived the project, designed and performed experiments, analyzed data, and wrote the paper. MG designed and performed experiments. ST and CM analyzed data. LV performed experiments. X-PZ generated and provided study mice. TH designed experiments. BP conceived the project, designed experiments, and edited the paper. All authors contributed to the article and approved the submitted version.

## Funding

This work was supported by the National Institutes of Health grants R01CA180699 and R01CA189524.

## Conflict of Interest

The authors declare that the research was conducted in the absence of any commercial or financial relationships that could be construed as a potential conflict of interest.

## Publisher’s Note

All claims expressed in this article are solely those of the authors and do not necessarily represent those of their affiliated organizations, or those of the publisher, the editors and the reviewers. Any product that may be evaluated in this article, or claim that may be made by its manufacturer, is not guaranteed or endorsed by the publisher.
