# Supplementary material for: Loss of Diacylglycerol Kinase α Enhances Macrophage Responsiveness
Source: Front Immunol. 2021 Nov 5;12:722469. doi: 10.3389/fimmu.2021.722469 (PMC8603347; doi:10.3389/fimmu.2021.722469)
Supplement: Supplementary file 1 [file DataSheet_1.docx]

Supplementary Material


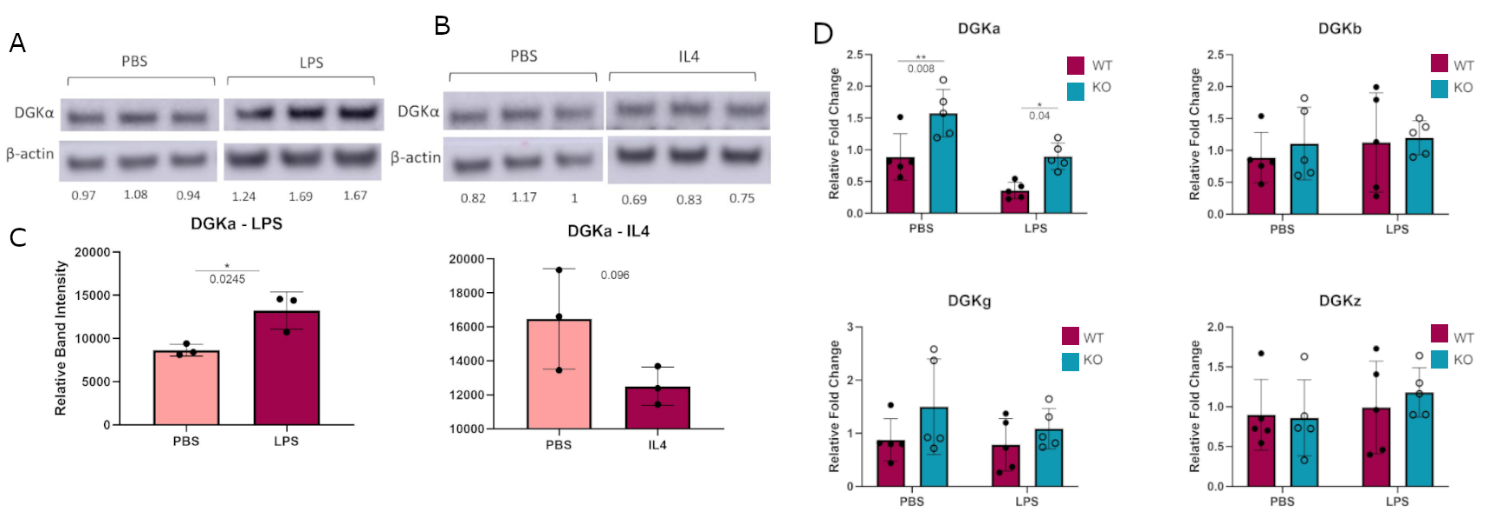


**Supplementary Figure 1. DGKα expression may be slightly altered by treatment with LPS and IL4, but expression of DGK family members is generally unaffected by knockout of *DGKa* or LPS treatment.** WT BMDMs were treated with PBS and LPS **(A)**, or PBS and IL4 **(B)** and DGKα protein expression was measured by immunoblot. Band intensities normalized to PBS treated group are indicated in the text below the bands. **(C)** Quantification of immunoblot band intensity. Error bars: SD. *p<0.05; student’s t-test. **(D)** WT and *Dgka^-/-^* BMDMs treated with PBS or LPS for 24h were assessed by qPCR for expression levels of type I DGKs a, b, and g, and type IV DGKz. DGKa KO was caused by a deletion between exon 9 to the intron between exons 16 and 17. The primer pair used to assess DGKa mRNA expression is prior to this deletion. Error bars: SD. *p<0.05, *p<0.01; Two-way ANOVA with Tukey post-hoc test.


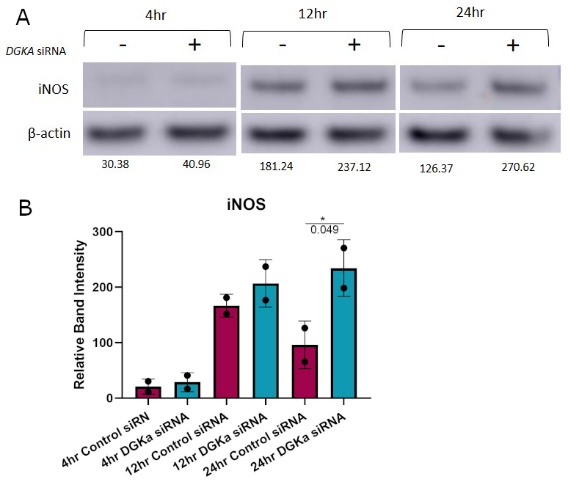


**Supplementary Figure 2. iNOS protein expression is not evident at early time points following LPS treatment of J774 macrophages. (A)** J774 cells transfected with control or *Dgka^-/-^* siRNA were treated with LPS for 4, 12, and 24h. Subsequent immunoblots probed for iNOS were quantified with band intensities relative to PBS treated controls (not shown) indicated in text below bands. **(B)** Quantification of immunoblot iNOS band intensity from two independent experiments. Error bars: SD. *p<0.05; Ordinary One-way ANOVA with Tukey post-hoc test.

**
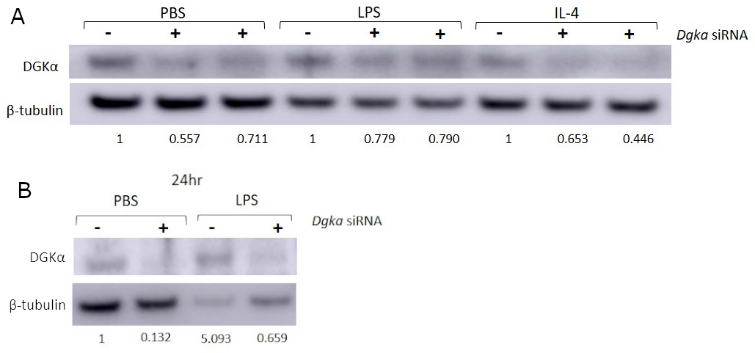
**

**Supplementary Figure 3. *Dgka* knockdown by transient siRNA transfection reduces DGKα protein expression. (A,B)** Immunoblot of whole cell lysates from transfected J774 cells probed for DGKα and β-tubulin. Relative band intensity normalized to respective control siRNA transfected cells per treatment group indicated in text below bands.


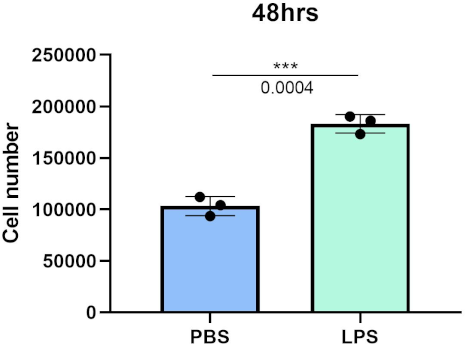


**Supplementary Figure 4. Increased cell numbers in LPS treated BMDMs after 48h.** WT BMDMs were treated with LPS for 48 hours and show increased cell numbers compared to PBS treated controls. Error bars: SD. ***p<0.001; Student’s t-test.


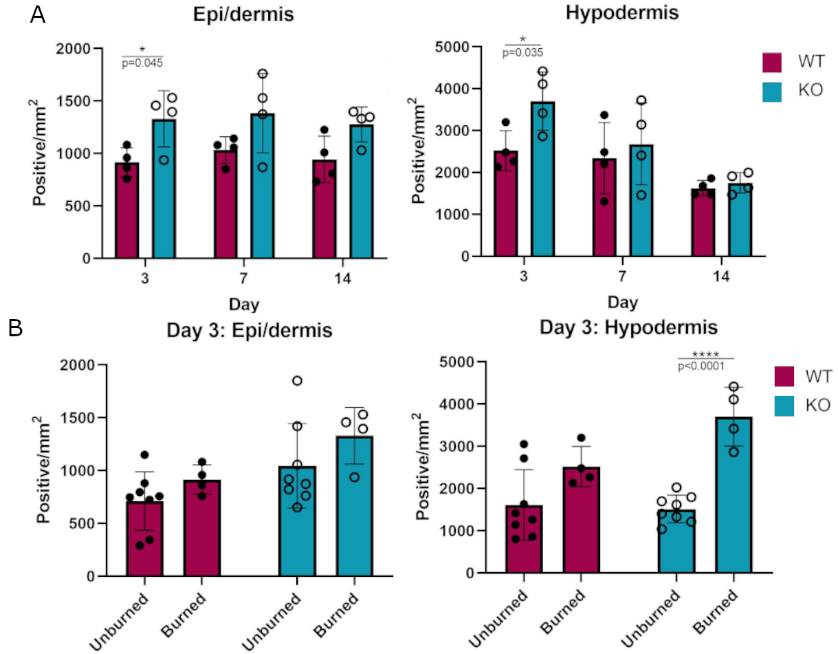


**Supplementary Figure 5. Iba1 staining in regions of burned/unburned skin of *Dgka^-/-^* versus WT mice over time demonstrate increased macrophage numbers in *Dgka^-/-^ mice*. (A)** In the epidermis/dermis of burned mice, the number of macrophages remains steady over time, while there is a spike in hypodermal macrophage infiltration at day 3. Which diminishes over time. **(B)** Comparison of burned vs. unburned skin do not show significant changes in macrophage numbers either in WT or KO mice at day 3. A significant increase over unburned macrophage counts is observed in the hypodermis of KO mice, not observed in WT. Each data point represents a single burn, with two burns per mouse per time-point for n=6 mice per group. Error bars: SD. *p<0.05, ****p<0.0001. Two-way ANOVA.


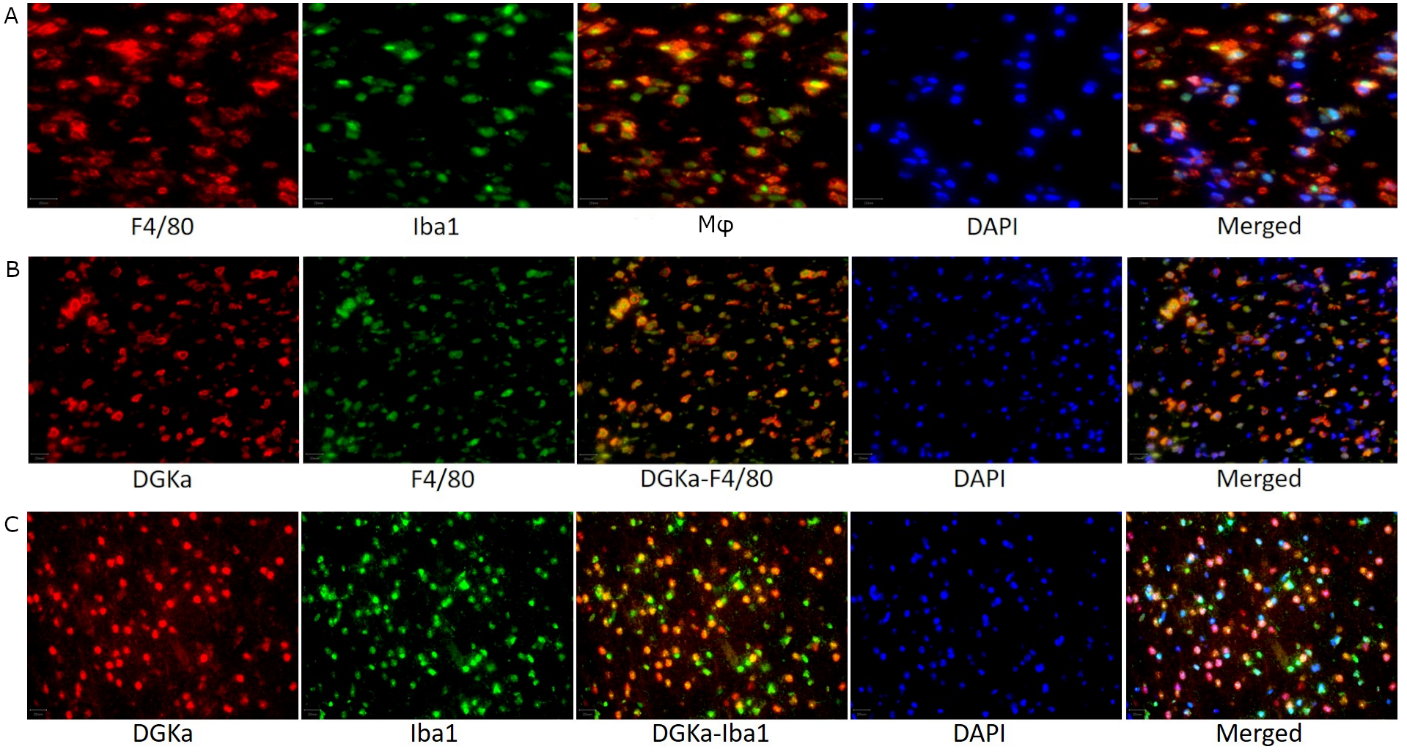


**Supplementary Figure 6. Colocalization of F4/80 and Iba1 in skin macrophages, and DGKa and colocalization with macrophages/microglia. (A)** Immunofluorescence (IF) staining and colocalization of F4/80 and Iba1 in mouse skin. **(B)** IF staining and colocalization of DGKa and F4/80 in mouse skin. **(C)** IF and colocalization of DGKa and Iba1 in mouse brain.
